# Structure and chronology of a star dune at Erg Chebbi, Morocco, reveals why star dunes are rarely recognised in the rock record

**DOI:** 10.1038/s41598-024-53485-3

**Published:** 2024-03-04

**Authors:** C. S. Bristow, G. A. T. Duller

**Affiliations:** 1 Department of Earth Sciences, University College London, London, UK; 2 Department of Geography and Earth Sciences, Aberystwyth University, Aberystwyth, UK

**Keywords:** Climate sciences, Planetary science, Solid Earth sciences

## Abstract

Star dunes are the tallest dunes on Earth and are amongst the larger and more spectacular aeolian landforms. Although they are widespread in modern sandy deserts, star dunes are rarely recognised in the rock record probably due to a lack of suitable sedimentary models. This paper presents a new sedimentary model for the structure of a star dune at Erg Chebbi in Morocco (Sahara Desert) on the basis of ground-penetrating radar (GPR) surveys. Individual sedimentary structures in star dunes are similar to those in linear or barchanoid dunes, likely leading to misidentification in the rock record. However, the suite of features described in this paper will permit identification of star dunes in future studies of the rock record. Optically stimulated luminescence (OSL) dating shows that accumulation of the Erg Chebbi star dune post-dates the end of the African Humid Period (AHP). At the base of the dune, there is an ~ 8000-year hiatus in the record. Since then, the dune has grown rapidly to create a 100 m high dune within the past 1000 years and is migrating towards the west. Changes in the cross-strata support the idea that star dune construction was accompanied by a change in the wind directions.

## Introduction

**Figure 1:**
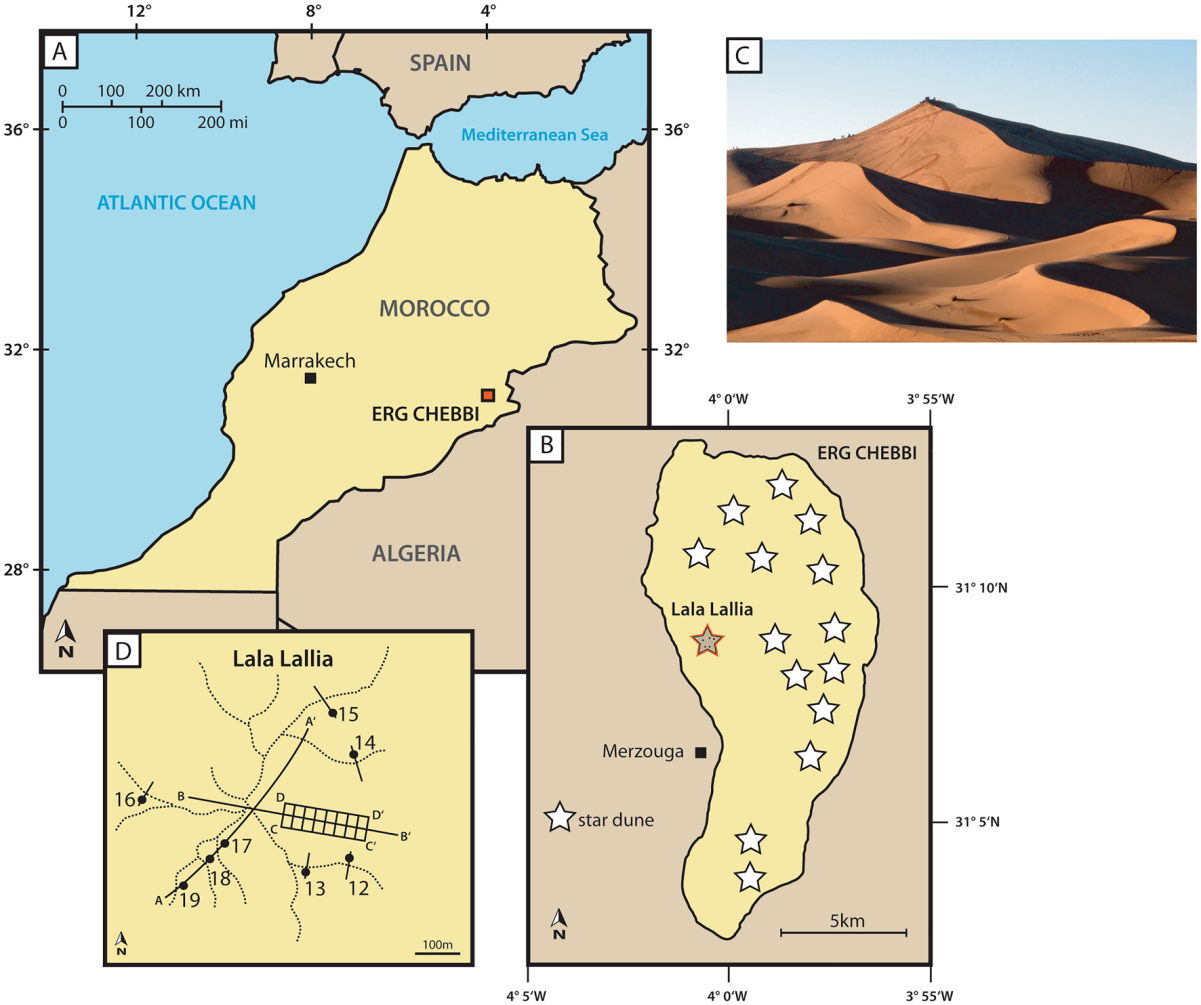
(**A**) Map of Morocco showing the location of Erg Chebbi. (**B**) Map of Erg Chebbi including the studied star dune Lala Lallia. (**C**) Photograph of the dune Lala Lallia from the South, looking north towards the dune crest. (**D**) Map of dune arms and crest lines (dashed lines) and GPR profiles (solid lines). Dots with numbers indicate OSL sample locations on the dune arms. Adobe Illustrator cs3 https://help.adobe.com/archive/en_US/illustrator/cs3/illustrator_cs3_help.pdf.

Star dunes, also sometimes known as pyramid dunes because of their shape (Fig. 1), are the largest and most complex type of desert sand dune. They have a pyramidal form and radiating arms and are widespread in modern deserts including sand seas in Africa, Arabia, China, and North America^1–3^, as well as on other bodies in the solar system such as Mars^4,5^ and Titan^6^. Within sand seas they are commonly the tallest dune^2,3,7^ and probably the tallest dunes on Earth^2,3^, reaching 300 m height in the Badain Jaran Desert in China^3,7,8^. Yet despite their large size and prominence within sand seas, or possibly because of their large size, star dunes are the least studied dune type^7–9^. Star dunes are found in areas with a variable wind regime and net sand accumulation^1,3,7,10^, and field observations and modelling of star dunes demonstrate that they grow vertically due to strong form-flow interactions^1,3,11–14^. Therefore, star dunes should be candidates for preservation in ancient aeolian sandstones^1^. However, with the exception of a Permian–Triassic example from Scotland^15^, star dune deposits have been rarely recognised in the rock record, possibly due to a lack of suitable sedimentary/stratigraphic models^16^.

This paper presents a new sedimentary model for star dune structures on the basis of a geophysical and geochronological investigation of a star dune in Erg Chebbi, Morocco. This work describes key sedimentary features and characteristics that can be searched for in the geological record. This work also determines the rate at which a star dune has accumulated, describes how the dune and its arms have developed, and proposes a dune chronology in relation to known Holocene climate changes in North Africa.

## Study site

Erg Chebbi is a small sand sea (18 km by 9 km) that includes reversing barchanoid dunes and star dunes, classified as complex star dunes^3^. The dune described here is known locally as Lala Lallia, which translates (from Berber) as the highest sacred point. On Google Earth™ it is shown as Hassilabied Gran Dune. It is a 100 m high, 700 m wide star dune with radiating arms (Fig. 1) that is located in the south of eastern Morocco close to the border with Algeria (31 08′49.46″N 04 00′34.04″W, Fig. 1). The wind regime is largely bimodal^17^ with a SW to NE 210°–240° “Sahel” wind known locally as the Sirocco, and an opposing NE-SW 45°–100° Chergui wind with a frequent, but weaker, easterly wind^18^ and supplementary text. Repeat surveys 16 months apart showed displacements perpendicular to the dune crestlines between 0 and 17 m but mostly less than 3 m^14^. The direction of displacement was extremely variable, changing direction between adjacent dune arms and along the arms, depending on the orientation to the wind. Analysis of spatial change during the same period shows that erosion and accumulation increase with elevation, meaning that the crest area is more dynamic than the lower arms^14^. This behaviour is likely due to wind acceleration up the dune, as observed with star dunes elsewhere^1,13,19^.

**Figure 2:**
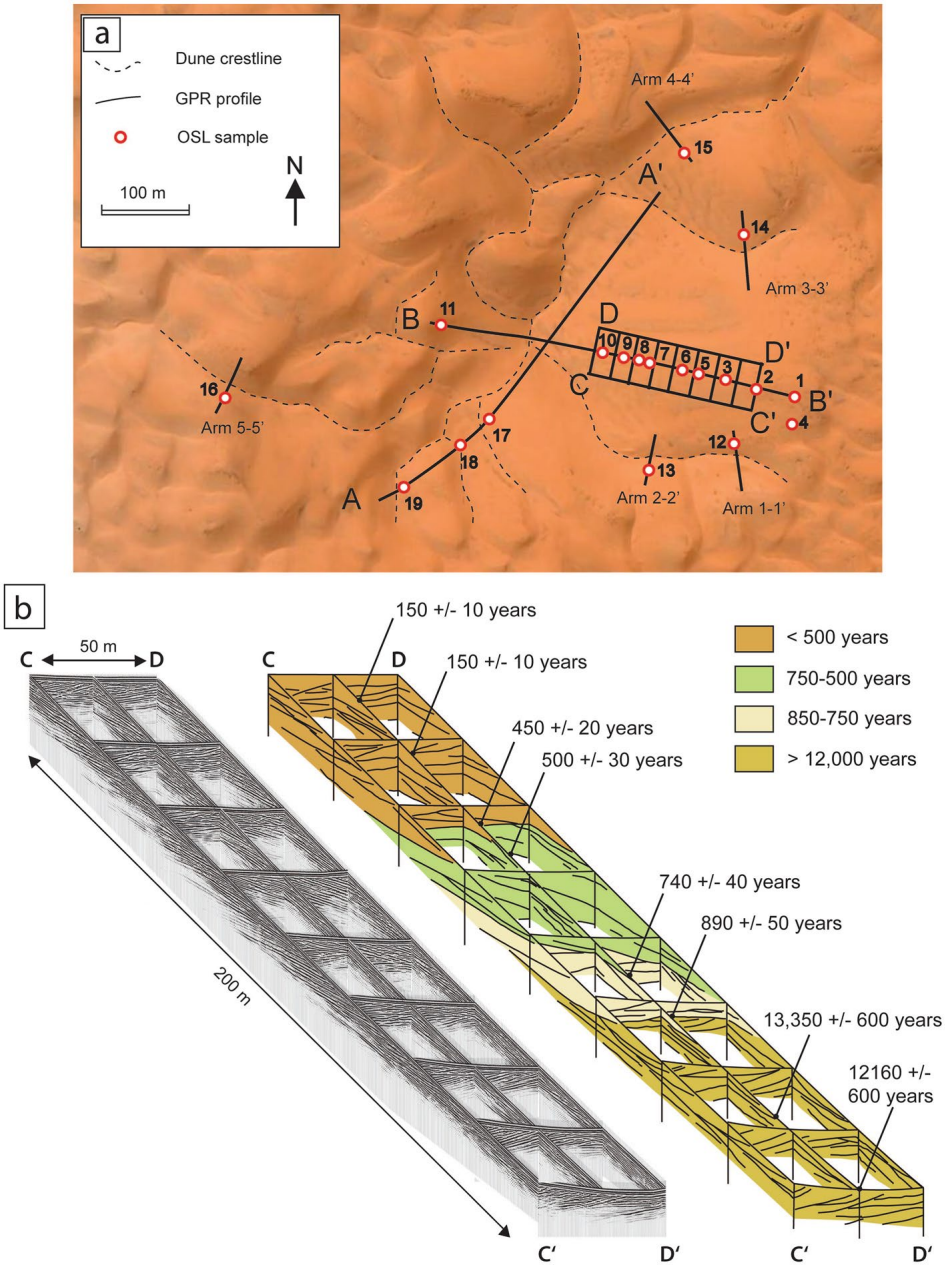
(**a**) Satellite image of Lala Lallia star dune from Google Earth 2023 Maxar Technologies with the dune crests marked by dashed lines, the GPR profiles as solid lines, and numbered circles indicating sample locations. (**b**) Grid of GPR profiles spaced 25 m apart on the eastern flank of the star dune with the age and location of optical dating of samples. The GPR profiles show sets of cross-strata within the dune dipping towards the southwest and northwest with bounding surfaces dipping gently from east to west showing that the dune has migrated from East to West. OSL ages decrease up the dune, consistent with vertical accumulation and East to West migration. The top 65 m of the dune have accumulated within the past 1000 years. The long profiles A–A′ and B–B′ as well as short profiles across the dune arms are illustrated in the supplementary material. Adobe Illustrator cs3 https://help.adobe.com/archive/en_US/illustrator/cs3/illustrator_cs3_help.pdf.

**Figure 3:**
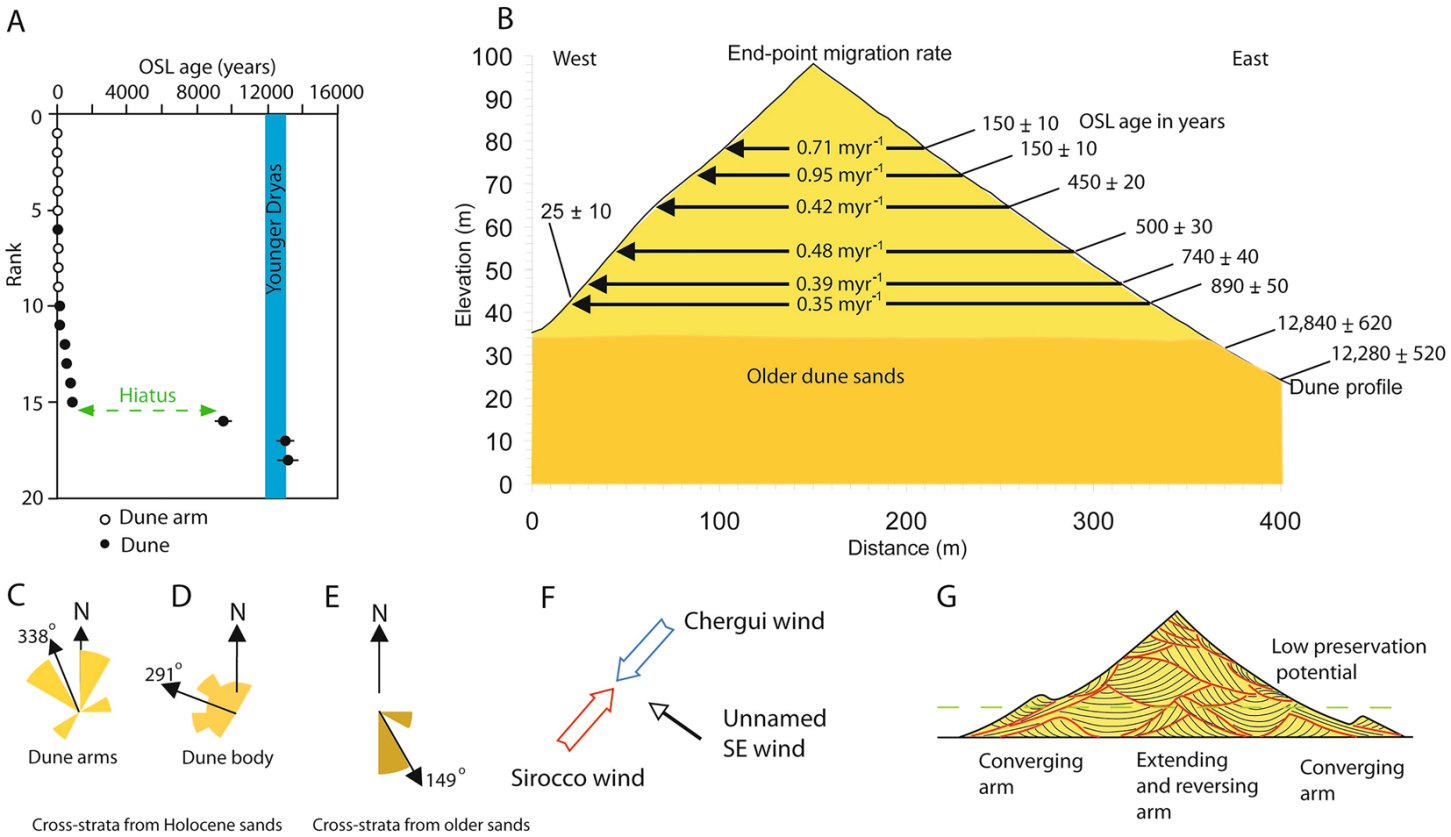
(**A**) Ranked OSL ages show dune accumulation during the past 1000 years following an 8000 year hiatus above samples from the early Holocene and Younger Dryas. With one exception, the dune arms are all younger than the main body of the dune. The exception is sample LL11 from the western, lee side of the dune. (**B**) Projecting the dune ages from the East (updrift) to the West (downdrift) gives estimates of the star dune migration rate around 0.5 m year^−1^. (**C**) Rose diagram showing that cross-strata from the dune arms have a wide dispersion. (**D**) Rose diagram of cross-strata from the past 1000 years dip towards the west and the northwest (mean 291°) perpendicular to the modern Chergui and Sirocco winds. (**E**) Rose diagram showing cross-strata from the early Holocene and Younger Dryas with dips to the east of South, indicating north and north-westerly winds. (**F**) Seasonal Chergui and Sirocco winds give a bimodal wind regime. (**G**) Model for star dune sedimentary structures; the red lines are bounding surfaces that separate sets of cross-strata within the dune and its arms. Note that the higher parts of the dune have low preservation potential and the lowest parts, beneath the dashed green line, are more likely to be preserved in the rock record. Adobe Illustrator cs3 https://help.adobe.com/archive/en_US/illustrator/cs3/illustrator_cs3_help.pdf.

Ground penetrating radar (GPR) profiles provide sections across the dune and its arms (supplementary data). A grid of GPR data was collected that gives three-dimensional insight into the dune strata (Fig. 2). Sample points for dating were selected on the basis of cross-cutting relations and superposition of strata imaged on the GPR profiles (Fig. 2 and Supplementary Figs. S4 and S5). Ten samples were collected for optical dating along the main axis of the dune (profile B–B′) in order to determine the rates of dune accumulation and migration using the optically stimulated luminescence (OSL) signal from quartz. An additional three samples were collected along the GPR profile A–A′, with five samples being used to constrain the age of the dune arms. At each OSL sample site, a pit 1 m deep was excavated for sampling, and from the same pits dip angle and dip direction of cross-strata were measured. The dip measurements are plotted as palaeocurrent roses in Fig. 3C–E. Cross-strata within the dune arms show a wide dispersion (Fig. 3C). In the pits for samples LL1–LL4, the strata are trough shaped and dip towards the south (Fig. 3E). Above 35 m elevation on the East side of the dune the strata are planar and dip towards the SW and NW (Fig. 3D). The wide dispersion of the cross-strata dip directions within the dune arms is consistent with the variable orientation of the dune arms. Along the profile B–B′ the measurements of cross-strata in the pits correspond with the orientation of reflections seen on the GPR profiles in Fig. 2.

## Chronology

**Figure 4:**
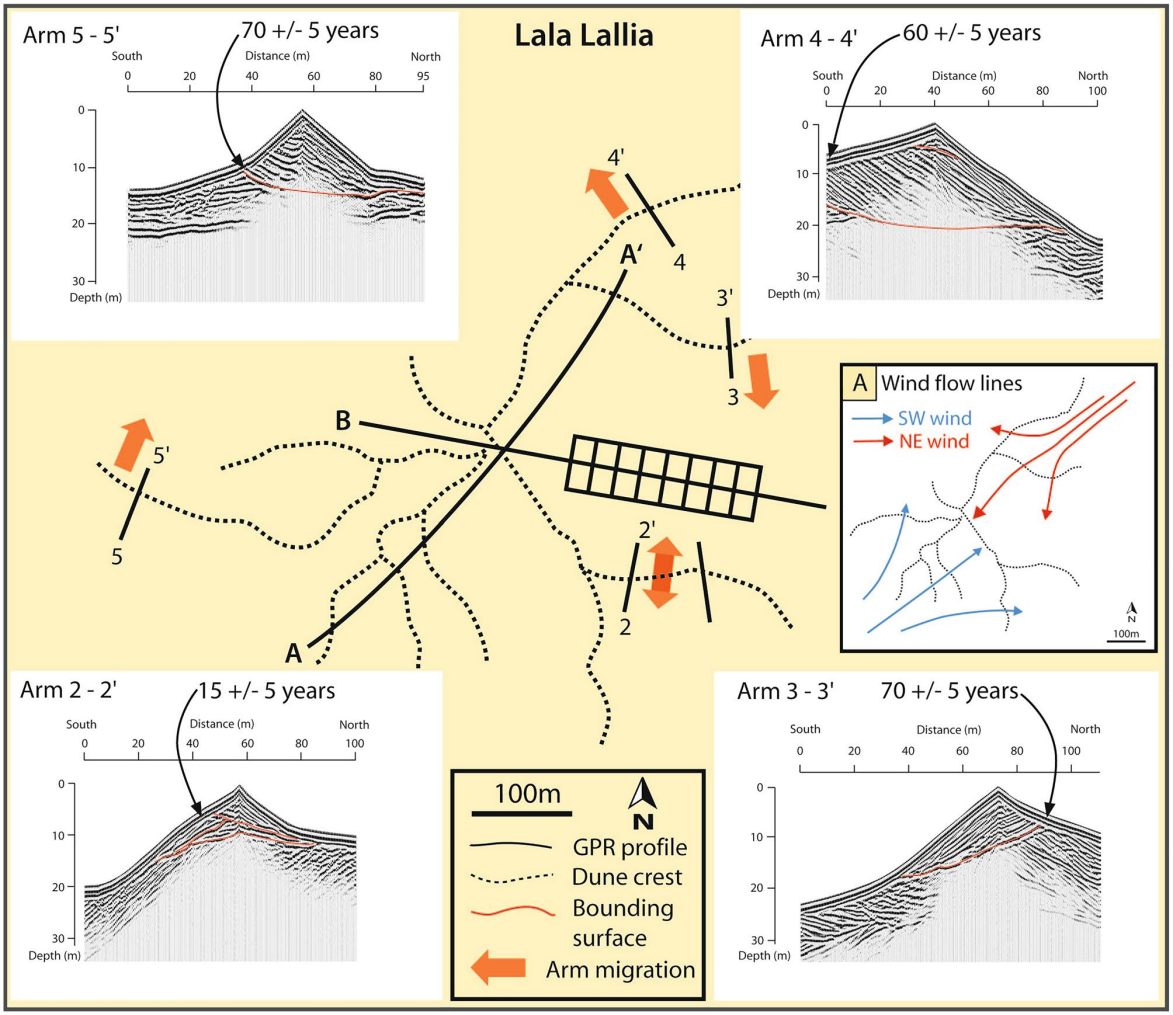
Sketch map showing star dune crest lines at the time the GPR data were collected (dashed lines) with four examples of GPR profiles across the dune arms as well as OSL ages from those arms. A. Inset shows interpreted wind flow lines diverted around the dune topography; blue arrows denote the SW Sirocco winds, and red arrows denote the NW Chergui winds. The GPR profiles show sets of cross-strata within each arm with an unconformity (erosion surface) at the base. The orange arrows indicate the direction of arm migration determined from the cross-strata imaged by the GPR profiles. Profile 2–2′ shows strata dipping in different directions, indicating that the arm has a reversing motion interpreted to be the product of the NW and SW winds. Profile 3–3′ images cross-strata dipping to the south above an unconformity (erosive base) that truncates older north-dipping strata. In contrast, profile 4–4′ that crosses an adjacent dune arm shows cross-strata dipping towards the northwest. Profile 5–5′ indicates arm migration towards the NNE. Adobe Illustrator cs3 https://help.adobe.com/archive/en_US/illustrator/cs3/illustrator_cs3_help.pdf.

Results of the optical dating yielded 19 ages (Fig. 3A; Table S2). Two samples from near the base of the dune yielded ages of 13,350 ± 600 years (sample LL3) and 12,160 ± 600 years (sample LL2), respectively. Another sample from the base of the dune to the east of the section described here yielded an age of 9530 ± 50 years (sample LL4). Optical dating also yielded 6 ages (samples LL5 through LL10) in a stratigraphic succession through the dune strata that give progressively younger ages towards the crest of the dune (Fig. 2). These 6 ages range from 890 ± 50 years at the base to 150 ± 10 years at the top. In addition, a sample on the western side of the dune (sample LL11) yielded an age of 25 ± 10 years (Fig. 3B and Table S2). OSL samples from the dune arms (samples LL12 through LL19) also show very young ages, ranging from 70 ± 5 years to 15 ± 5 years (Table S2 and Fig. 4).

The two oldest ages (LL2, LL3) are coincident, within uncertainties, with the Younger Dryas event (which is dated at 12,896–11,703 years b2k (before 2000 CE)^20^). Above the sediments that yielded the older ages (LL2, LL3, LL4), there appears to be an ~ 8000 year hiatus in net sediment accumulation, above which are the sediments that yielded the age of 890 ± 50 years (sample LL5) and younger ages. The youngest age along profile B–B′ (25 ± 10 years) is from the western side of the dune, indicating modern sand accumulation on the lee slope and confirming the net westerly migration of the dune.

## Migration

Rates of migration can be calculated using an end-point migration rate method^21^. Using this method, the calculated rates of the past 890 years vary between 0.35 and 0.95 m year^−1^, with an average of 0.55 m year^−1^ and an apparent increase in migration rate towards the present (Fig. 3B). The crest of a 20 m high star dune in China is reported to have migrated 16 m over a period of 13 years^13^, suggesting an annual migration rate of 1.2 m year^−1^ for a smaller star dune. There is limited data on migration rates of other large dunes, but the migration rates of the Lala Lallia star dune are much greater than those calculated for linear dunes in Namibia^22^, and megadunes in China^23^, but similar to the rate of 0.56 m year^−1^ reported for a 55 m high barchan dune in Peru^24^.

## Dune strata

Ground Penetrating Radar (GPR) revealed internal sedimentary structures (inclined GPR reflections) that are interpreted as sets of cross-strata separated by lower angle bounding surfaces (Figs. 2 and 4). The grid of GPR profiles on the east side of the dune shows inclined reflections that dip towards the northwest and northeast with low-angle bounding surfaces dipping towards the west.

Rose diagrams showing the orientation of dune strata are interpreted as indicating palaeowind directions (Fig. 3D,E). The older dune strata with ages from the YD and early Holocene dip towards the SSE (mode 150°) indicating winds from NNW. In contrast, the younger strata less than 1000 years old dip towards the southwest and northwest with a mean of 291° (Fig. 3C). These observations are supported by the GPR profiles, which show trough cross-strata overlain by planar strata with bimodal dips towards the NW and SW (Fig. 2). The older trough cross-strata are interpreted as the product of barchanoid dunes migrating from north to south towards 150° driven by NNW winds blowing south from the Atlas Mountains. The younger dune strata, less than 1000 years old, do not appear to be aligned with either the NE Chergui or the SW Sirocco winds. Instead, the younger cross-strata appear to suggest winds from the east, and the mean of 291° is roughly perpendicular to the dominant modern winds (Fig. 3F). If considering topographic steering or deflection of winds around the dune (Fig. 3F), then the dune arms are roughly perpendicular to the dominant wind directions, thus satisfying the maximum gross bedform normal rule^25^. Dune arms aligned NE and SW are oriented parallel to the Chergui and Sirocco winds as predicted by^26^. However, arms oriented towards the west are explained by secondary flow converging in the lee of the dune. The bimodal dips and orientation perpendicular to the reversing Chergui and Sirocco winds are consistent with the existing conceptual model for star dune development^1^ where the arms of a star dune develop from secondary flow around a reversing dune. Some dune arms are aligned with the wind as predicted by numerical modelling^26^, other arms are perpendicular to the Chergiu and Sirocco winds apparently maximising transport across rather than along the dune arms. Although it has been suggested that the two modes might be sequential^26^, field observations suggest that both modes can operate at the same time. The net migration towards the west preserves north-westerly and south-westerly dipping strata produced by wind deflection across the arms of the star dune as noted in a separate study of a star dune in China^13^. The westward migration and vertical accretion of sets of cross-stratification are reinforced by the optical ages, which are progressively younger up the dune and from East to West (Fig. 2). The younger ages at the crest of the dune indicate increased mobility due to the wind speed-up towards the dune crest combined with a smaller cross-sectional area at the dune crest. Enhanced wind speed and reduced cross-sectional area result in a dynamic dune crest with m of crestline displacement during a storm^14^.

The arms of the star dune are sinuous, branching, and most often asymmetric in cross-section with one face steeper than the other. The relative steepness of the faces is an indication of their exposure to the wind and the resulting migration around the flanks of the star dune. This migration is recorded by sets of cross-strata preserved within the dune arms with the steeper face on the lee side, and cross-strata dipping towards the direction of migration. The GPR profiles in Fig. 4 show the strata within the dune arms as well as an unconformity (erosion surface) at the base of each arm. The migration direction recorded by the cross-strata varies around the dune. On the northern side of the dune, cross-strata within the arm indicate migration towards the northwest (4–4′ Fig. 4). On the eastern side of the dune, the GPR profiles show cross-strata dipping towards the south (3–3 Fig. 4) and to the north and south (2–2 Fig. 4) indicating that the arms have migrated towards the south (3–3′), and a bimodal north–south dip indicating arm reversals (2–2′ Fig. 4). On the west side of the dune, the strata within the arm dip towards the north (5–5′). When each arm is viewed with respect to the prevailing NE and SW winds, a pattern can be identified that is interpreted to be a response to the relative exposure of each arm (Fig. 4A). The arms on the north and east flanks of the dune are relatively sheltered from the SW winds but fully exposed to the NE winds and consequently show dips towards the NW (4–4′) and towards the South (3–3′), as the north-westerly winds are deflected around the flanks of the dune. Conversely, an arm on the west side of the dune that is primarily exposed to the SW winds and is relatively sheltered from the NE winds has migrated towards the north (5–5′ Fig. 4). An arm that is equally exposed to the NE and SW winds contains strata that dip towards the north and the south due to crest reversal as the arm moves back and forth under alternating winds (2–2′ Fig. 4).

The base of each arm is marked by an unconformity (erosion surface) that truncates underlying dune strata and is down-lapped by cross-strata from the migrating arms (Fig. 4). The direction of arm migration and orientation of the dune strata vary around the dune indicating that the arms migrate independently from the star dune as they wrap around the flanks of the dune in response to their relative exposure to the prevailing winds. The arms converge towards the dune crest helping to build the star dune.

## Palaeoclimate

The unconformity (a hiatus in dune sand accumulation and/or erosion of dune sand) identified in this study is marked in the field by scattered pottery fragments, rhizoliths, and fulgurites on the east side of the dune [31°08′49″N 04°00′24″W]. The rhizoliths indicate the presence of vegetation that is interpreted to have stabilized the dune for at least part of this period. The pottery indicates human occupation of the dune, and the fulgurites (which are formed by lightning strikes on sand dunes) indicate the occurrence of thunderstorms. Together these features indicate wetter conditions when the dune was likely to have been stabilized by vegetation.

The timing and duration of the hiatus in sand dune accumulation and/or erosion of dune sand during the early and middle Holocene have implications for palaeoclimate conditions and possible climate forcing, on the basis of limited marine and terrestrial climate proxy data from similar latitudes (31°N) in Morocco. The northwards extent of the West African monsoon (WAM) during the African humid period (AHP) is likely to have had a strong influence on aeolian activity within the Sahara^27,28^. The offshore sediment record at 31°N indicates a decrease in fluvial sediment input and a contemporary increase in aeolian dust at the onset of the Younger Dryas around 12.7–12.4 kyr BP^29^. This change in the aeolian input to the offshore record is interpreted to indicate a probable change in wind intensity at that time^29^, which is consistent with the dune ages reported here although the cross-strata suggest that there might also have been a change in wind direction. Inferred annual precipitation derived from leaf wax isotopes in marine sediments at 31°N suggests a strong but relatively restricted duration for the AHP at this latitude, with the humid period starting around 10 kyr BP and ending around 6.5 kyr BP^30^, although the end of the AHP is diachronous^31^. A strong monsoon, possibly accompanied by winter rainfall^30^, could have resulted in stabilisation (by vegetation) of the sand dunes in Erg Chebbi, and is likely to be the primary control on the observed gap in the dune sand luminescence ages. The δ^18^O record from the Wintimdouine speleothem (31°N; Morocco) shows a multi-millennial ‘heavy-light-heavy’ pattern through the Holocene, consistent with the northward expansion of the WAM with a hiatus interpreted as drought conditions about 4 to 3.3 kyr BP^32^. The OSL ages from the star dune post-date this drought and the age attributed to the end of the AHP^30^. The lag between the onset of aridity and the accumulation of the star dune could be attributed to a number of possibilities. It is most likely that the dunes were active during this period but sediments were not preserved (i.e., no net accumulation). There are several examples where dunes were thought to have been active during a certain time interval, but luminescence ages from the sediment report only the most recent remobilization of the sediments^27^. It is also possible that the dune accumulation record within the star dune Lalla Lallia relates to the time taken for the dune to migrate into its current location. An alternative explanation is that the dunes at Erg Chebbi were stabilised by an elevated water table that remains elevated to this day and is exploited by subterranean drainage channels called khettaras (Qanat) that are used to irrigate palm trees and gardens along the edge of the erg^33^. Another hypothesis is that there is a lag between the onset of aridity and the mobilisation of sufficient sand to construct a star dune.

The results of this study indicate rapid star dune construction building a 100 m high dune in less than 1000 years and migrating west at around 0.5 m year^−1^, confirming that star dunes migrate. Despite this movement, they can retain a record of climate change and paleowind behaviour. In this case the dune strata record significant changes in palaeowinds during the last 12 ka. The dune strata record northerly winds during the Younger Dryas. A cessation in aeolian accumulation occurred during the early Holocene, resulting in an unconformity (stabilisation surface) marked by fulgurites, rhizoliths, and pottery (representing thunderstorms, vegetation of the dunes, and human occupation, respectively). There are parallels here with the dune chronology in the Gran Desierto, Mexico, where star dunes have developed above an unconformity (stabilisation surface) on older, Late Pleistocene to Early Holocene sand dunes^34^. At Erg Chebbi, dune sediment accumulation resumed during the late Holocene with a change in wind regime where NE and SW winds contributed to construction of the star dune with arms that are both perpendicular to and parallel with the NE and SW winds, suggesting that two modes of dune formation can co-exist. The net westward migration of the star dune may be influenced by the regional easterly trade winds across the Sahara.

The model of star dune sedimentary structures includes extending and reversing dune arms, converging arms that wrap around the dune, multiple bounding surfaces, and large, 3.5 to 7.5 m thick, sets of cross-strata formed by the main dune slipface (Fig. 3G). Because sand dunes are preserved from the bottom-up, the strata from the arms that extend in the downdrift direction, as well as the arms that wrap around the dune and converge beneath the main body of the dune, have a much higher preservation potential than the sediments of the star dune peak. The base of a star dune may be marked by an unconformity that is an erosional contact on earlier dune deposits, as observed here at Erg Chebbi and in the Gran Desierto, Mexico the preservation of extending and reversing dune arms that resemble linear dunes^35,36^, as well as converging dune arms covered by larger sets of cross-strata, could be mistaken for strata deposited by barchanoid dunes^37^. Thus, star dune deposits in the rock record could be mistaken for deposits of other types of dune. The data presented in this paper provide a sedimentary model for the identification of ancient star dune deposits in the sedimentary record. Furthermore, the model presented here incorporates sedimentary structures found within the main body of the dune as well as the dune arms. This new model is different from a model proposed for star dunes in California (USA)^16^ that emphasised coarse grained low-angle strata around the base of star dunes. Due to their large size, the identification of ancient star dune deposits will require study of extensive outcrops in order to distinguish the combination of depositional elements described here.

### Supplementary Information

Supplementary Figure S4.

Supplementary Tables.

Supplementary Information.

## Data Availability

OSL data and processed GPR data generated and analysed during this study are included in this published article and its supplementary information files. Raw, unprocessed GPR data files generated during the current study are available from the corresponding author on reasonable request.

## References

[1] Lancaster N (1989). The dynamics of star dunes: An example from the Grand Desierto, Mexico. Sedimentology.

[2] Lancaster N (1989). Star dunes. Prog. Phys. Geogr..

[3] Goudie AS, Goudie AM, Viles HA (2021). The distribution and nature of star dunes: A global analysis. Aeolian Res..

[4] Edgett KS, Blumberg DG (1994). Star and linear dunes on Mars. Icarus.

[5] Silvestro S, Fenton LK, Michaels TI, Valdez A, Ori GG (2012). Interpretation of the complex dune morphology on Mars: Dune activity, modelling and a terrestrial analogue. Earth Surf. Proc. Landf..

[6] Ewing RC, Hayes AG, Lucas A (2015). Sand dune patterns on Titan controlled by long-term climate cycles. Nat. Geosci..

[7] Dong Z, Wang T, Wang X (2004). Geomorphology of the megadunes in the Badain Jaran Desert. Geomorphology.

[8] Chen JS, Li L, Wang JY, Barry DA (2004). Groundwater maintains dune landscape. Nature.

[9] Tan L, Zhang W, Bian K, Zhishan A, Zu R, Qu J (2016). Numerical simulation of three-dimensional wind flow patterns over a star dune. J. Wind Eng. Ind. Aerodyn..

[10] Wasson RJ, Hyde R (1983). The factors determining desert dune type. Nature.

[11] Wilson IC (1972). Aeolian bedforms—Their development and origins. Sedimentology.

[12] McKee ED (1982). Sedimentary structures in dunes in the Namib Desert, South West Africa. Geol. Soc. Am. Spec. Pap..

[13] Wang T, Zhang W, Dong Z, Qu J, Jing Z, Wang W, Feng J (2005). The dynamic characteristics and migration of a pyramid dune. Sedimentology.

[14] Herzog M, Anders K, Höfle B, Bubenzer O (2022). Capturing complex star dune dynamics-repeated highly accurate surveys combining multitemporal 3D topographic measurements and local wind data. Earth Surf. Process. Landf..

[15] Clemmensen LB, Frostick LE, Read I (1987). Complex star dunes and associated aeolian bedforms, Hopeman Sandstone (Permo-Triassic), Moray Firth Basin, Scotland. Desert Sediments: Ancient and Modern.

[16] Neilson J, Kocurek G (1987). Surface processes, deposits and development of star dunes: Dumont dune field, California. Geol. Soc. Am. Bull..

[17] Puy A, Herzog M, Escriche P, Marouche A, Oubana Y, Bubenzer O (2018). Detection of sand encroachment patterns in desert oases. The case of Erg Chebbi (Morocco). Sci. Total Environ..

[18] Mainguet M, Cossus L, Balkema AA (1980). Sand circulation in the Sahara: Geomorphological relations between the Sahara Desert and its margins. Palaeoecology of Africa and the Surrounding Islands.

[19] Zang W, Qu J, Tan L, Jing Z, Bian K, Niu Q (2016). Environmental dynamics of a star dune. Geomorphology.

[20] Rasmussen SO (2006). A new Greenland ice core chronology for the last glacial termination. J. Geophys. Res..

[21] Bristow CS, Lancaster N, Duller GAT (2005). Combining ground penetrating radar surveys and optical dating to determine dune migration in Namibia. J. Geol. Soc..

[22] Bristow CS, Duller GAT, Lancaster N (2007). Age and dynamics of linear dunes in the Namib desert. Geology.

[23] Zhao H, Li B, Wang XF, Cohen TJ, Fan YX, Yang HY, Wang KQ, Sheng YW, Zhan SA, Li SH, Wang T, Wang XL, Chen FH (2023). Evolution and migration of the highest megadunes on Earth. Glob. Planet. Change.

[24] Simons FS (1956). A note on Pur-Pur Dune, Viru Valley, Peru. J. Geol..

[25] Rubin DM, Hunter RE (1987). Bedform alignment in directionally varying flows. Science.

[26] Zhang D, Narteau C, Rozier O, Courech du Pont S (2012). Morphology and dynamics of star dunes from numerical modelling. Nat. Geosci..

[27] Swezey C (2001). Eolian sediment responses to late Quaternary climate changes: Temporal and spatial patterns in the Sahara. Palaeogeogr. Palaeoclimatol. Palaeoecol..

[28] Bristow CS, Armitage SJ (2016). Dune ages in the sand deserts of the southern Sahara and the Sahel. Quatern. Int..

[29] Holz C, Stuut J-BW, Henrich R, Meggers H (2007). Variability in terrigenous sedimentation processes off northwest Africa and its relation to climate changes: Inferences from grain-size distributions of a Holocene marine sediment record. Sediment. Geol..

[30] Tierney JE, Pausata FSR, deMenocal PB (2017). Rainfall regimes of the Green Sahara. Sci. Adv..

[31] Shanahan TM, McKay NP, Hughen KA, Overpeck JT, Otto-Bliesner B, Heil CW, King J, Scholz CA, Peck J (2015). The time-transgressive termination of the African Humid Period. Nat. Geosci..

[32] Sha L, Brahim YA, Wassenburg JA, Yin J, Peros M, Cruz FW, Cai Y, Li H, Du W, Zhang H, Edwards RL, Cheng H (2019). How far north did the African Monsoon fringe expand during the African Humid Period? Insights from Southwest Moroccan speleothems. Geophys. Res. Lett..

[33] Garcia-Rodriguez M, Anton L, Martinez-Santos P (2014). Estimating groundwater resources in remote desert environments by coupling geographic information systems with groundwater modelling (Erg Chebbi, Morocco). J. Arid Environ..

[34] Beveridge C, Kocurek G, Ewing RC, Lancaster N, Morthekai P, Singhvi AK, Mahan SA (2006). Development of spatially diverse and complex dune-field patterns: Gran Desierto Dune Field, Sonora, Mexico. Sedimentology.

[35] Bristow CS, Bailey SD, Lancaster N (2000). The sedimentary structure of linear sand dunes. Nature.

[36] Scotti AA, Veiga GD (2019). Sedimentary architecture of an ancient linear megadune (Barremian, Neuquen Basin): Insights into the long-term development and evolution of aeolian linear bedforms. Sedimentology.

[37] McKee ED (1966). Structures of dunes at White Sands National Monument, New Mexico, and comparison with dunes from other selected areas. Sedimentology.

