# Supplementary figures and images for: Structure and chronology of a star dune at Erg Chebbi, Morocco, reveals why star dunes are rarely recognised in the rock record
Source: Sci Rep. 2024 Mar 4;14:4464. doi: 10.1038/s41598-024-53485-3 (PMC10909956; doi:10.1038/s41598-024-53485-3)

# GPR profiles across star dune Lala Lallia, Erg Chebbi, Morocco

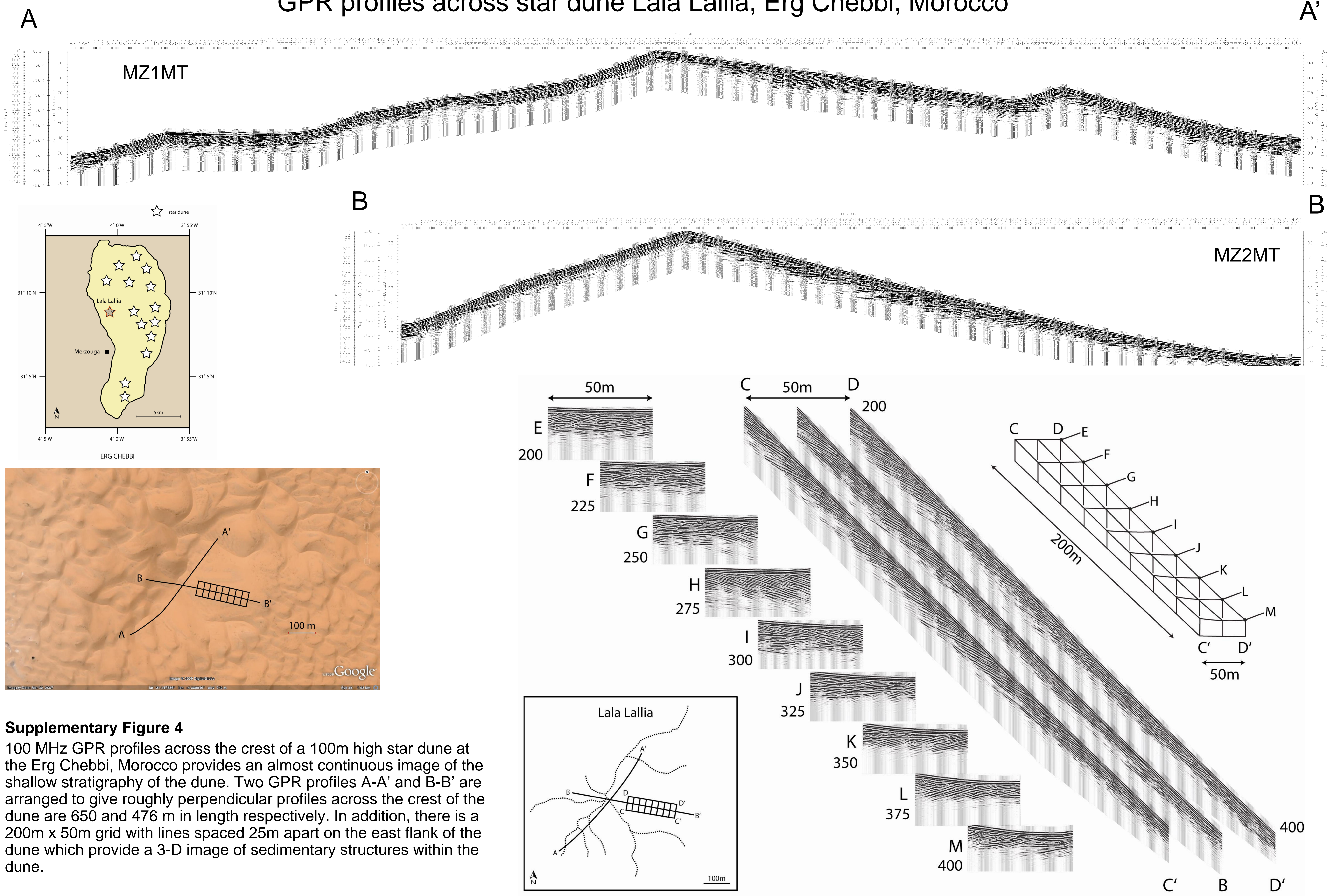

Supplement: Supplementary file 1 — Supplementary Figure S4. [file 41598_2024_53485_MOESM1_ESM.pdf]
