# Supplementary material for: Structure and chronology of a star dune at Erg Chebbi, Morocco, reveals why star dunes are rarely recognised in the rock record
Source: Sci Rep. 2024 Mar 4;14:4464. doi: 10.1038/s41598-024-53485-3 (PMC10909956; doi:10.1038/s41598-024-53485-3)
Supplement: Supplementary file 2 — Supplementary Tables. [file 41598_2024_53485_MOESM2_ESM.docx]

**Supplementary Table S1**

Dosimetry data for luminescence samples collected at Lala Lallia. Depth at which samples were collected is given, along with the calculated concentrations of U, Th and K in the bulk sediment determined with thick source alpha counting and GM-beta counting. The contribution of beta, gamma and cosmic radiation to the total dose rate received by quartz grains used for luminescence dating are also shown. Details are given in the Supplementary Information text.

| Sample | Depth (m) | U  (ppm) | Th  (ppm) | K  (%) | Beta Dose (Gy/ka) | Gamma Dose (Gy/ka) | Cosmic Dose (Gy/ka) | Total Dose (Gy/ka) |
| --- | --- | --- | --- | --- | --- | --- | --- | --- |
| Aber156/LL1 | 1.00 | 0.45 ± 0.06 | 1.67 ± 0.20 | 0.22 ± 0.02 | 0.23 ± 0.01 | 0.18 ± 0.01 | 0.21 ± 0.02 | 0.65 ± 0.03 |
| Aber156/LL2 | 1.00 | 0.53 ± 0.06 | 1.33 ± 0.18 | 0.22 ± 0.02 | 0.24 ± 0.01 | 0.17 ± 0.01 | 0.21 ± 0.02 | 0.65 ± 0.03 |
| Aber156/LL3 | 1.10 | 0.51 ± 0.06 | 1.70 ± 0.20 | 0.22 ± 0.02 | 0.24 ± 0.01 | 0.19 ± 0.01 | 0.21 ± 0.02 | 0.66 ± 0.03 |
| Aber156/LL4 | 1.10 | 0.37 ± 0.07 | 2.10 ± 0.22 | 0.27 ± 0.02 | 0.26 ± 0.01 | 0.20 ± 0.01 | 0.21 ± 0.02 | 0.71 ± 0.03 |
| Aber156/LL5 | 1.05 | 0.45 ± 0.05 | 1.28 ± 0.16 | 0.31 ± 0.02 | 0.28 ± 0.01 | 0.18 ± 0.01 | 0.21 ± 0.02 | 0.71 ± 0.03 |
| Aber156/LL6 | 1.00 | 0.39 ± 0.05 | 1.39 ± 0.16 | 0.20 ± 0.02 | 0.20 ± 0.01 | 0.15 ± 0.01 | 0.21 ± 0.02 | 0.60 ± 0.03 |
| Aber156/LL7 | 1.00 | 0.41 ± 0.06 | 1.91 ± 0.21 | 0.25 ± 0.02 | 0.25 ± 0.01 | 0.19 ± 0.01 | 0.21 ± 0.02 | 0.68 ± 0.03 |
| Aber156/LL8 | 1.10 | 0.45 ± 0.06 | 1.50 ± 0.18 | 0.24 ± 0.02 | 0.24 ± 0.01 | 0.18 ± 0.01 | 0.21 ± 0.02 | 0.66 ± 0.03 |
| Aber156/LL9 | 1.00 | 0.46 ± 0.06 | 1.57 ± 0.19 | 0.31 ± 0.02 | 0.29 ± 0.01 | 0.20 ± 0.01 | 0.21 ± 0.02 | 0.72 ± 0.03 |
| Aber156/LL10 | 1.20 | 0.40 ± 0.05 | 1.51 ± 0.17 | 0.30 ± 0.02 | 0.27 ± 0.01 | 0.19 ± 0.01 | 0.21 ± 0.02 | 0.69 ± 0.03 |
| Aber156/LL11 | 0.85 | 0.36 ± 0.05 | 1.32 ± 0.15 | 0.16 ± 0.02 | 0.18 ± 0.01 | 0.14 ± 0.01 | 0.22 ± 0.02 | 0.56 ± 0.03 |
| Aber156/LL12 | 1.00 | 0.60 ± 0.06 | 1.34 ± 0.18 | 0.16 ± 0.02 | 0.20 ± 0.01 | 0.17 ± 0.01 | 0.21 ± 0.02 | 0.61 ± 0.03 |
| Aber156/LL13 | 1.00 | 0.63 ± 0.07 | 1.68 ± 0.22 | 0.20 ± 0.02 | 0.24 ± 0.01 | 0.19 ± 0.01 | 0.21 ± 0.02 | 0.67 ± 0.03 |
| Aber156/LL14 | 0.70 | 0.50 ± 0.05 | 1.39 ± 0.18 | 0.29 ± 0.02 | 0.27 ± 0.01 | 0.19 ± 0.01 | 0.22 ± 0.02 | 0.71 ± 0.03 |
| Aber156/LL15 | 0.85 | 0.36 ± 0.06 | 1.66 ± 0.19 | 0.25 ± 0.02 | 0.24 ± 0.01 | 0.18 ± 0.01 | 0.22 ± 0.02 | 0.66 ± 0.03 |
| Aber156/LL16 | 1.00 | 0.36 ± 0.03 | 1.23 ± 0.11 | 0.19 ± 0.01 | 0.19 ± 0.01 | 0.14 ± 0.01 | 0.21 ± 0.02 | 0.57 ± 0.03 |
| Aber156/LL17 | 0.85 | 0.12 ± 0.05 | 1.98 ± 0.16 | 0.25 ± 0.02 | 0.22 ± 0.01 | 0.17 ± 0.01 | 0.22 ± 0.02 | 0.63 ± 0.03 |
| Aber156/LL18 | 1.10 | 0.24 ± 0.04 | 1.53 ± 0.14 | 0.29 ± 0.02 | 0.25 ± 0.01 | 0.17 ± 0.01 | 0.21 ± 0.02 | 0.65 ± 0.03 |
| Aber156/LL19 | 0.75 | 0.20 ± 0.05 | 1.99 ± 0.16 | 0.29 ± 0.02 | 0.25 ± 0.01 | 0.18 ± 0.01 | 0.22 ± 0.02 | 0.68 ± 0.03 |

Notes:

1. Dose rates were calculated assuming a water content of 3±2% for all samples, and also a grain size of 180-211 µm for all samples except LL16 whose grain size was 180-250µm
2. Cosmic ray dose rate was calculated using the current burial depth and the equation of Prescott and Hutton (1994)
3. Internal alpha dose rate of 0.030±0.005 Gy/ka has been included in the total dose rate. See text for details.

**Supplementary Table S2**

Dose rate to quartz grains from Supplementary Table 1, along with the number of aliquots measured to determine equivalent dose, the average equivalent dose, and ages calculated for each sample. All ages were calculated as years before the date of measurement which was 2009 AD for samples LL1-LL11, and 2013 AD for samples LL12-19, but then the difference of 4 years was added to LL1-LL11, so that all ages are given as years before 2013 AD. Ages are rounded to the nearest five years for ages below 100 years old, and to the nearest 10 years for older ages.

| Sample | Dose Rate (Gy/ka) | Number of aliquots | Equivalent Dose (Gy) | Age (a) |
| --- | --- | --- | --- | --- |
| Aber156/LL1 | 0.65 ± 0.03 | 17 | 0.03 ± 0.00 | 45 ± 5 |
| Aber156/LL2 | 0.65 ± 0.03 | 21 | 7.91 ± 0.22 | 12,160 ± 600 |
| Aber156/LL3 | 0.66 ± 0.03 | 63 | 8.85 ± 0.16 | 13,350 ± 600 |
| Aber156/LL4 | 0.71 ± 0.03 | 21 | 6.72 ± 0.26 | 9,530 ± 540 |
| Aber156/LL5 | 0.71 ± 0.03 | 17 | 0.63 ± 0.03 | 890 ± 50 |
| Aber156/LL6 | 0.60 ± 0.03 | 18 | 0.44 ± 0.02 | 740 ± 40 |
| Aber156/LL7 | 0.68 ± 0.03 | 20 | 0.33 ± 0.01 | 500 ± 30 |
| Aber156/LL8 | 0.66 ± 0.03 | 21 | 0.30 ± 0.01 | 450 ± 20 |
| Aber156/LL9 | 0.72 ± 0.03 | 21 | 0.11 ± 0.01 | 150 ± 10 |
| Aber156/LL10 | 0.69 ± 0.03 | 21 | 0.10 ± 0.01 | 150 ± 10 |
| Aber156/LL11 | 0.56 ± 0.03 | 19 | 0.01 ± 0.00 | 25 ± 10 |
| Aber156/LL12 | 0.61 ± 0.03 | 39 | 0.02 ± 0.01 | 35 ± 10 |
| Aber156/LL13 | 0.67 ± 0.03 | 35 | 0.01 ± 0.00 | 15 ± 5 |
| Aber156/LL14 | 0.71 ± 0.03 | 13 | 0.05 ± 0.01 | 70 ± 5 |
| Aber156/LL15 | 0.66 ± 0.03 | 34 | 0.04 ± 0.00 | 60 ± 5 |
| Aber156/LL16 | 0.57 ± 0.03 | 17 | 0.04 ± 0.00 | 70 ± 5 |
| Aber156/LL17 | 0.63 ± 0.03 | 19 | 0.02 ± 0.00 | 25 ± 5 |
| Aber156/LL18 | 0.65 ± 0.03 | 17 | 0.01 ± 0.00 | 20 ± 5 |
| Aber156/LL19 | 0.68 ± 0.03 | 18 | 0.02 ± 0.00 | 25 ± 5 |
