# Supplementary material for: Structure and chronology of a star dune at Erg Chebbi, Morocco, reveals why star dunes are rarely recognised in the rock record
Source: Sci Rep. 2024 Mar 4;14:4464. doi: 10.1038/s41598-024-53485-3 (PMC10909956; doi:10.1038/s41598-024-53485-3)
Supplement: Supplementary file 3 — Supplementary Information. [file 41598_2024_53485_MOESM3_ESM.docx]

**Structure and chronology of a star dune at Erg Chebbi, Morocco, reveals why star dunes are rarely recognised in the rock record**

**Supplementary Text**

**Wind regime**

Wind data for Morocco is sparse but there is a meteorological station at Errachidia airport which is 95 km to the NNE of Erg Chebbi. At this airport, the dominant wind directions are fairly consistent through the year and confirm local reports of two dominant wind directions from the northeast ‘Chergui’ and southwest ‘Sirocco’ with a subsidiary easterly wind. The occurrence of north easterly and south westerly winds has been recorded at Tafilalt, approximately 40 km NW of Erg Chebbi, by Kabiri et al. (2003) ^S1^ and the wind regime at Erg Chebbi has been described as bimodal (Puy et al. 2018) ^s2^. A wind rose diagram for Erg Chebbi using the coordinates latitude 31.1470 degrees North and longitude -4.0080 degrees West has been compiled using data of ERA5 hourly wind speed and direction at 10 m above ground, from 1-1-2003 through 31-12-2022 (https://cds.climate.copernicus.eu/cdsapp#!/dataset/reanalysis-era5-single-levels?tab=overview).

The wind rose diagram (Figure S1) is divided into 16 segments. The radius indicates the duration of winds from a given direction as a percentage, whereas the colours blue to red indicate the wind strength in m/s. The wind rose diagram shows that the wind blows most often from the northeast and the southwest, confirming previous reports of a bimodal wind regime ^S1, S2^. Winds from the northeast are the most frequent, occurring more than 12% of the year on average, whereas winds from the southwest are the strongest (Figure S1). The sand rose calculations are based on the Fryberger method (Fryberger and Dean 1979) ^s3^, but all calculations are in metric units, with a transport threshold of 6.2 m/s at 10 m height. The sand rose diagram shows the bimodal trend (Figure S2) with a drift potential (DP) of 18 and a resultant drift potential (RDP) of 3, and a resultant drift direction towards the NNE 24 degrees. The resultant drift direction of 24 degrees is at odds with the dune migration direction as interpreted from the GPR data, which suggest dune migration towards the west. However, the effectively opposite northeast and southwest transport vectors are nearly in balance and the value of the resultant drift direction RDD is so low that with such a very low RDP/DP value the RDD becomes essentially arbitrary (influenced by very small vectors from other directions). The difference between the observed dune migration to the west and the calculated sand drift towards the NNE might, in part, be due to the threshold wind velocity used in the sand rose calculation above. Field experience and geomorphology including transverse incipient dunes on the east slope (Figure 7 of Herzog et al. 2022) demonstrate that the east winds are active on the upper half of the dune. East winds are shown on the wind rose diagram (Figure S1) but at an elevation of 10 m they rarely exceed the transport threshold velocity of 6.2 m/s. However, the calculations for the sand rose diagram do not take account of wind acceleration up the dune. Such acceleration has been reported on star dunes in China (Zhang et al 2000, Wang et al. 2005), and has been observed in the field at Erg Chebbi. The calculated value for the resultant drift is very low because the NE and SW winds are almost equal and opposite. In contrast, the wind rose diagram shows easterly and southeast winds almost 30% of the time, whereas opposing winds from the west and northwest are rare (Figure S1). It is likely that wind acceleration up the star dune effectively reduces the transport threshold velocity and as a result the easterly winds are more important whereas the northeast and southwest winds continue to cancel each other out, thus shifting the resultant drift direction towards the west as recorded by the dune strata.

**Ground-Penetrating Radar (GPR)**

Ground-penetrating radar (GPR) data were collected using a Pulse Ekko PE 100 with 1000 volt transmitter, 100 MHz antennas, in a parallel broadside configuration with a separation of 1 m, a step size of 0.5 m, and 32 stacks. Data were collected along profiles across the dunes with tape measures laid across the surface of the dune to constrain the position of each point on the ground. The locations for the ends of the lines were recorded with a hand-held global positioning system (GPS) and the elevations along the profiles were measured using a Sokkia total station reduced to a local temporary benchmark. Data processing included dewow, down-the-trace filters, and trace-to-trace filters with an automatic gain control maximum 200 and corrections for elevation interpolated between topographic survey measurements every 5 m as well as at breaks of slope.

**OSL dating**

Samples for optical stimulated luminescence (OSL) dating were collected in opaque plastic tubes pushed and (or) hammered into the face of hand-excavated soil pits at an average depth of 1 m (Supplementary Table S1). The samples were analysed in the Aberystwyth Luminescence Research Laboratory (ALRL) to determine the period of time since the last exposure of the sediment to daylight. Once in the laboratory, each sample was split. One split was used to determine the radiation dose rate and the other split was used for luminescence measurements. The portion used for dosimetry was oven-dried and then milled to a fine powder. Thick source alpha counting (TSAC) was used to determine the concentration of uranium (U) and thorium (Th) in each sample, and the infinite matrix beta dose rate was measured using a Risø GM-25-5 beta counter. The potassium (K) concentration in the sediment was determined by combining the TSAC results and the beta counting (Supplementary Table S1). The beta dose received by samples during burial was calculated by correcting the infinite matrix beta dose rate for the effects of grain size (180-211 µm for all samples except Aber156/LL16, which was 180-250 µm) and water content. A water content of 3±2% was used in all calculations. The gamma dose rate was calculated from the concentrations of U, Th, and K given in Supplementary Table S1, and corrected for the effect of water content. Quartz was isolated from the sediment and used for luminescence measurements (see below). Previous studies have found that sedimentary quartz grains commonly contain small traces of U and Th and that the alpha dose delivered by these radionuclides should be accounted for. This situation is especially important where the external dose rate is low, as is the case at the Erg Chebbi study site. An internal alpha dose rate of 0.030 ± 0.005 Gy/ka was added to the quartz dose rates, on the basis of taking the mean of values measured by Jacobs et al. (2008)^S4^, Clarkson et al. (2017)^S5^, and Tooth et al. (2022)^S6^. The final contribution to the total dose rate is the cosmic dose rate, which was calculated from the sample depth using the equations given by Prescott and Hutton (1994) ^S7^. Dose rates and ages were calculated using the Dose Rate and Age Calculator (DRAC; Durcan et al 2015) ^S8^.

The portion of each sample used for luminescence measurement was treated with 10 vols H_2_O_2_ to remove organics and 10% HCl to remove carbonates, although very little reaction was seen in either reagent for this suite of samples. Samples were then dried and sieved to isolate grains between 180-211 µm (180-250 µm for sample Aber156/LL16). To isolate quartz grains, the samples were then separated on the basis of density using solutions of sodium polytungstate at densities of 2.62 and 2.70 g.cm^-3^. Samples were then placed in 40% hydrofluoric acid (HF) for an hour to remove any residual feldspars, and to etch the alpha-irradiated outer 10 µm of the quartz grains. At the end of the etching, samples were rinsed in concentrated hydrochloric acid (HCl) to remove any fluorides, and then neutralised. The final stage of the sample preparation for luminescence measurements was to re-sieve at 180 µm to remove any fragments of feldspar.

The purified quartz was mounted on 9.8 mm diameter aluminium discs. “Medium” sized aliquots, approximately 5 mm in diameter and consisting of about 500 grains, were prepared because it was assumed that these samples would be well bleached at deposition (Duller 2008) ^S9^. Equivalent dose was calculated using the single aliquot regenerative dose (SAR) method using the optically stimulated luminescence (OSL) signal. OSL measurements were made on a Risø TL/OSL reader equipped with blue-light emitting diodes (470 Δ 20 nm) for optical stimulation, and an EMI 9635QA photomultiplier tube (PMT) for detection of the OSL. Two 2.5 mm thick Hoya U-340 filters were placed in front of the PMT to shield it from the stimulation light. A strontium (Sr)/yttrium (Y) beta source mounted on the reader was used for artificial irradiation. Measurement of the OSL signal was for 100 s while holding the sample at 125°C to prevent retrapping at the defect responsible for the 110 °C thermoluminescence peak. A typical OSL decay curve and SAR dose response curve are shown in Supplementary Figure S3.

Appropriate thermal treatments for these samples were determined by undertaking a preheat test on samples Aber156/LL3 and LL8. In each case 24 aliquots were measured, with three replicate measurements at each preheat temperature (160, 180, 200, 220, 240, 260, 280, and 300 °C held for 10 s), and using a cutheat of 160 °C. Consistent values of equivalent dose could be obtained using any of these preheat treatments for both samples. A preheat of 200 °C was selected for dating because for these two samples it showed the lowest scatter between replicate aliquots.

For each of the 19 samples (Aber156/LL1 to LL19), at least 24 medium aliquots were measured. At least twenty-one of these aliquots were used for dating, and at least three were used to perform a dose recovery test. All aliquots were screened using standard criteria to judge the reliability of the data (Duller 2003; Roberts 2008) ^S10, S11^. Aliquots were only accepted if they had recycling ratios within 10% of unity, if they had infrared (IR) OSL depletion ratios within 10% of unity, gave a test dose signal at least three times above the standard deviation of the background, and did not suffer from recuperation. Recuperation was assessed by the luminescence signal measured following the zero dose measurement in the SAR sequence. This signal was expressed as a percentage of the natural signal for samples where the equivalent dose was above 5 Gy (Aber156/LL2, LL3 and LL4), and expressed as an absolute dose (by extrapolating the dose response curve to the x axis) for the remaining samples where the equivalent dose was less than 1 Gy. The acceptance thresholds were 5% of the natural signal and an absolute dose of 0.05 Gy. The number of aliquots that passed these acceptance criteria varied from 63 to 13 (Supplementary Table S2). Individual estimates of equivalent dose were combined to calculate a mean, with the uncertainty calculated as the standard deviation (“error”). These values were then used for age calculation (Supplementary Table S2). Dose recovery experiments on 3 aliquots from each sample (giving a total of 57 aliquots) gave a ratio of the measured dose divided by the known laboratory dose of 1.01±0.01, demonstrating the suitability of the SAR parameters for accurately measuring dose in these samples. All the luminescence ages obtained for these samples are in stratigraphic order.

**Sand accumulation**

Volume calculations assume that the dune is a square-based pyramid and use the formula for the volume of a pyramid where the volume is equal to one third of the base area times the height. This calculation yields a volume of 3.4 million cubic meters, using the height above the unconformity (stratigraphic hiatus). The mass of sand that has accumulated during the past 850 years can be calculated assuming a density for dry sand of 1.6 tonnes/m3. The use of this assumption yields a total mass of 5.44 million tonnes of packed dry sand, which amounts to an annual rate of accumulation of 6,400 tonnes per annum.

**Supplementary text references**

S1. Kabiri, L., Boudad, L., Krimou, A., Khardi, A., Elmrani, L., 2003, Preliminary study of continental dunes in southeastern Morocco (Tafilalt, Morocco). Science and Global Change/Drought, 14, p.149-156.

S2. Puy, A., Herzog, M., Pedro, E., Marouche, A., Oubana, Y., and Bubenzer, O., 2018. Detection of sand encroachment patterns at desert oases. Science of the Total Environment 642, 241-249. https://doi.org/10.1016/j.scitotenv.2018.05.343 et al.

S3. Fryberger, S.G., and Dean, G., 1979. Dune forms and wind regime. In McKee, E.D., Ed. A Study of Global Sand Seas. US Geological Survey Professional Paper 1052, p.137-169.

S4. Jacobs Z., Roberts R.G., Galbraith R.F., Deacon H.J., Grun R., Mackay A.W., Mitchell P., Vogelsang R. and Wadley L. (2008). Ages for the Middle Stone Age of Southern Africa: Implications for human behavior and dispersal. Science 322: 733-735.

S5. Clarkson C., Jacobs Z., Marwick B., Fullagar R., Wallis L., Smith M., Roberts R.G., Hayes E., Lowe K., Carah X., Florin S.A., McNeil J., Cox D., Arnold L.J., Hua Q., Huntley J., Brand H.E.A., Manne T., Fairbairn A., Shulmeister J., Lyle L., Salinas M., Page M., Connell K., Park G., Norman K., Murphy T. and Pardoe C. (2017). Human occupation of northern Australia by 65,000 years ago. Nature 547: 306-310.

S6. Tooth S., McCarthy T.S., Duller G.A.T., Assine M.L., Wolski P. and Coetzee G. (2022). Significantly enhanced mid Holocene fluvial activity in a globally important, arid-zone wetland: the Okavango Delta, Botswana. Earth Surface Processes and Landforms 47(3): 854-871.

S7. Prescott, J. R. and Hutton, J. T. (1994). Cosmic ray contributions to dose rates for luminescence and ESR dating: large depths and long-term time variations. Radiation Measurements 23: 497-500.

S8. Durcan J.A., King G.E. and Duller G.A.T. (2015) DRAC: dose rate and age calculator for trapped charge dating. Quaternary Geochronology 28: 54-61.

S9. Duller, G.A.T. (2008). Single grain optical dating of Quaternary sediments: why aliquot size matters in luminescence dating. Boreas 37: 589-612.

S10. Duller, G.A.T. (2003). Distinguishing quartz and feldspar in single grain luminescence measurements. Radiation Measurements 37: 161-165.

S11. Roberts, H. M. (2008). The development and application of luminescence dating to loess deposits: a perspective on the past, present, and future. Boreas 37: 483-507.

**Supplementary Figure S1**

Wind rose for Erg Chebbi constructed with ERA5 hourly wind speed and direction at 10 m above ground, from 1-1-2003 through 31-12-2022 The wind rose diagram illustrates the dominant NE and SW wind directions as well as lower velocity winds from the E and SE.
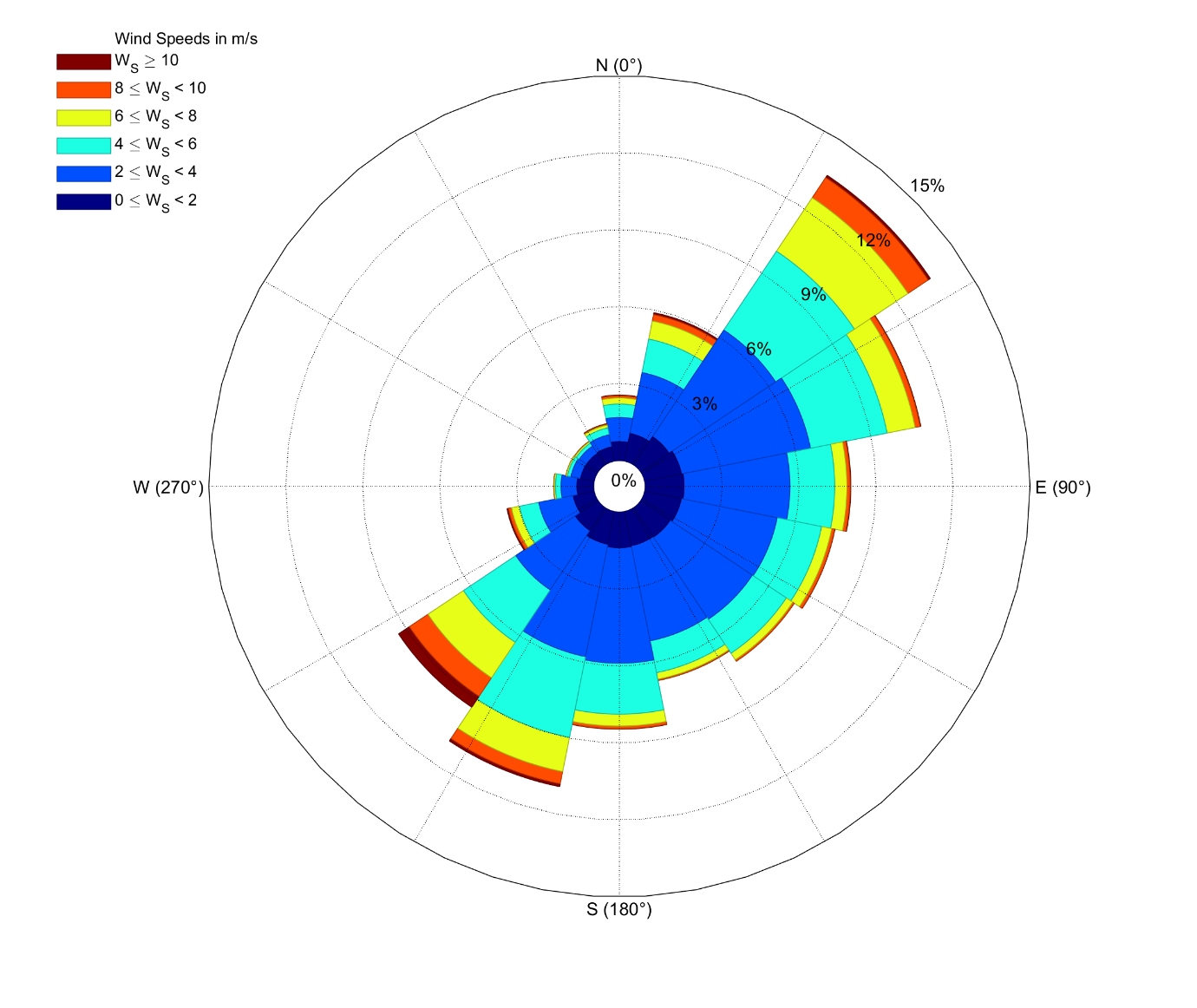


**Supplementary Figure S2**


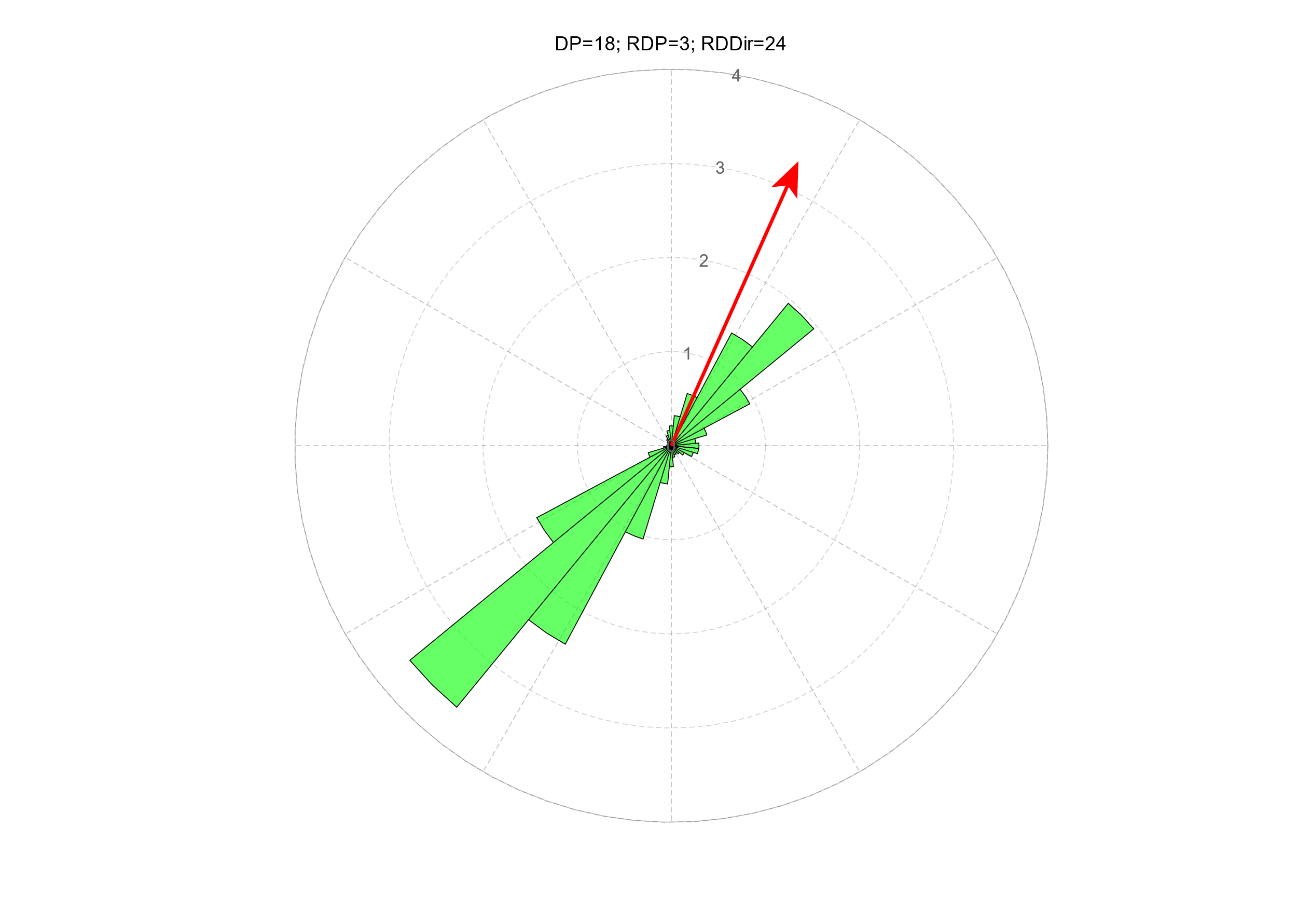


The sand rose diagram shows the bimodal trend (Figure S2) with a drift potential (DP) of 18, a resultant drift potential (RDP) of 3, and a resultant drift direction towards the NNE 24 degrees.

**Supplementary Figure S3**

Example of a typical dose response curve for sample Aber156/LL5. The equivalent dose for this aliquot is 0.58±0.03 Gy. The inset shows the natural OSL decay curve measured for this aliquot exhibiting a very rapid initial drop in the signal. Note the logarithmic scale on the y-axis of the inset.

**Supplementary Figure S4 Poster PDF**

100 MHz GPR profiles across the crest of a 100 m high star dune at the Erg Chebbi (Morocco) provide an almost continuous image of the shallow stratigraphy of the dune. The two GPR profiles A-A’ and B-B’, which are arranged to give roughly perpendicular profiles across the crest of the dune, are 650 and 476 m in length respectively. In addition, there is a 200 m x 50 m grid with lines spaced 25 m apart on the east flank of the dune. This grid provides a 3-D image of sedimentary structures within the dune (Figure 2).

Supplementary Figure S5

Map showing location of all of the OSL sample locations across the dune and its arms. Adobe Illustrator cs3 https://help.adobe.com/archive/en_US/illustrator/cs3/illustrator_cs3_help.pdf


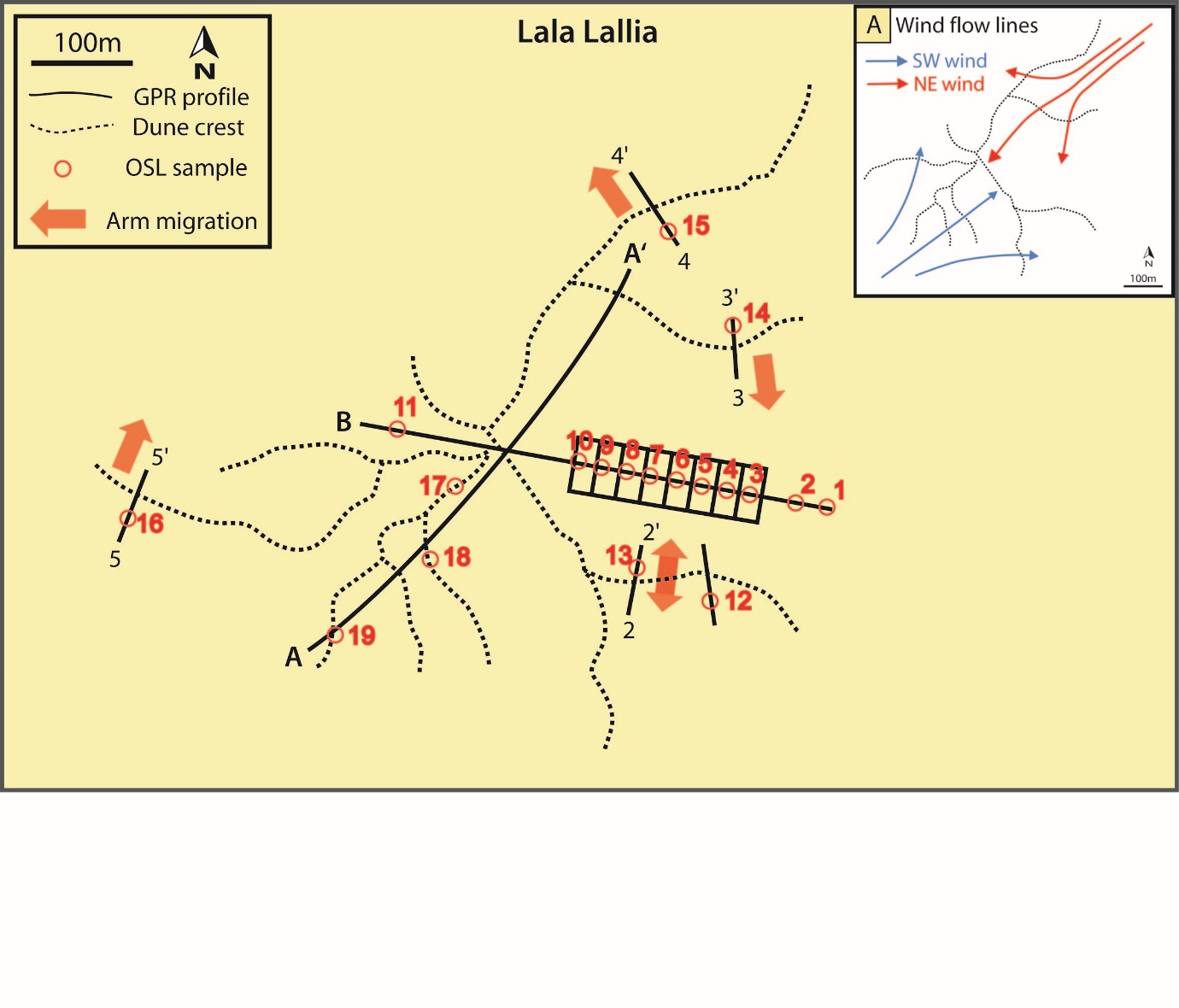


Supplementary figure S6

Model of star dune sedimentary structures derived from this study showing dune arms that resemble linear dunes and converging, encircling arms beneath large sets of trough cross-strata with a bimodal orientation. Note that the higher parts of the dune have low preservation potential and the lowest parts, beneath the dashed green line, are more likely to be preserved in the rock record because sand dunes are preserved from the bottom-up. Strata from the arms that extend in the downdrift direction, as well as the arms that wrap around the dune and converge beneath the main body of the dune, have a much greater preservation potential than the characteristic peak of the dune. The red lines are bounding surfaces that separate sets of cross strata from the dune and its arms (Figure 3 G)


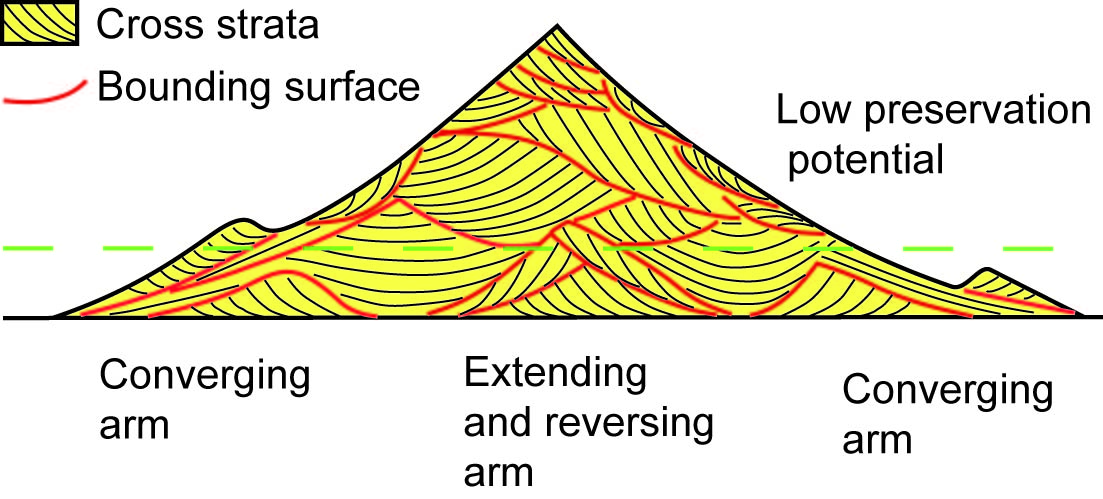


**Supplementary tables in landscape format**
